# The *RAB39B* p.G192R mutation causes X-linked dominant Parkinson’s disease

**DOI:** 10.1186/s13024-015-0045-4

**Published:** 2015-09-24

**Authors:** Ignacio F. Mata, Yongwoo Jang, Chun-Hyung Kim, David S. Hanna, Michael O. Dorschner, Ali Samii, Pinky Agarwal, John W. Roberts, Olga Klepitskaya, David R. Shprecher, Kathryn A. Chung, Stewart A. Factor, Alberto J. Espay, Fredy J. Revilla, Donald S. Higgins, Irene Litvan, James B. Leverenz, Dora Yearout, Miguel Inca-Martinez, Erica Martinez, Tiffany R. Thompson, Brenna A. Cholerton, Shu-Ching Hu, Karen L. Edwards, Kwang-Soo Kim, Cyrus P. Zabetian

**Affiliations:** Veterans Affairs Puget Sound Health Care System, Seattle, WA USA; Department of Neurology, University of Washington School of Medicine, Seattle, WA USA; Molecular Neurobiology Laboratory, Department of Psychiatry and Program in Neuroscience, McLean Hospital/Harvard Medical School, Belmont, MA USA; Department of Psychiatry and Behavioral Sciences, University of Washington, Seattle, WA USA; Department of Pathology, University of Washington, Seattle, WA USA; Booth Gardner Parkinson’s Care Center, Evergreen Hospital Medical Center, Kirkland, WA USA; Virginia Mason Medical Center, Seattle, WA USA; Department of Neurology, University of Colorado, Denver, USA; Department of Neurology, University of Utah, Salt Lake City, UT USA; Parkinson’s Disease Research, Education, and Clinical Center, Portland Veterans Affairs Medical Center, Portland, OR USA; Department of Neurology, Oregon Health and Science University, Portland, OR USA; Department of Neurology, Emory University School of Medicine, Atlanta, GA USA; Department of Neurology and Rehabilitation Medicine, University of Cincinnati, Cincinnati, OH USA; Division of Neurology at Greenville Health System and the University of South Carolina Medical School-Greenville, Greenville, SC USA; Samuel Stratton Veterans Affairs Medical Center, Albany, NY USA; Movement Disorder Center, Department of Neurosciences, University of California, San Diego, CA USA; Lou Ruvo Center for Brain Health, Cleveland Clinic, Cleveland, OH USA; Neurogenetics Research Center, Instituto Nacional de Ciencias Neurologicas, Lima, Peru; Department of Epidemiology, University of California, Irvine, CA USA

## Abstract

**Objective:**

To identify the causal gene in a multi-incident U.S. kindred with Parkinson’s disease (PD).

**Methods:**

We characterized a family with a classical PD phenotype in which 7 individuals (5 males and 2 females) were affected with a mean age at onset of 46.1 years (range, 29-57 years). We performed whole exome sequencing on 4 affected and 1 unaffected family members. Sanger-sequencing was then used to verify and genotype all candidate variants in the remainder of the pedigree. Cultured cells transfected with wild-type or mutant constructs were used to characterize proteins of interest.

**Results:**

We identified a missense mutation (c.574G > A; p.G192R) in the *RAB39B* gene that closely segregated with disease and exhibited X-linked dominant inheritance with reduced penetrance in females. The mutation occurred in a highly conserved amino acid residue and was not observed among 87,725 X chromosomes in the Exome Aggregation Consortium dataset. Sequencing of the *RAB39B* coding region in 587 familial PD cases yielded two additional mutations (c.428C > G [p.A143G] and c.624_626delGAG [p.R209del]) that were predicted to be deleterious *in silico* but occurred in families that were not sufficiently informative to assess segregation with disease. Experiments in PC12 and SK-N-BE(2)C cells demonstrated that p.G192R resulted in mislocalization of the mutant protein, possibly by altering the structure of the hypervariable C-terminal domain which mediates intracellular targeting.

**Conclusions:**

Our findings implicate RAB39B, an essential regulator of vesicular-trafficking, in clinically typical PD. Further characterization of normal and aberrant RAB39B function might elucidate important mechanisms underlying neurodegeneration in PD and related disorders.

**Electronic supplementary material:**

The online version of this article (doi:10.1186/s13024-015-0045-4) contains supplementary material, which is available to authorized users.

## Background

Parkinson’s disease (PD) is the second most common neurodegenerative disorder and though approximately 20 % of patients report a family history of the disease, kindreds that display clear Mendelian inheritance are rare. However, mutations in several genes have been shown to result in clinically typical autosomal dominant (*SNCA*, *LRRK2*, *VPS35*, *DNAJC13*) or recessive (*PARK2*, *PINK1*, *PARK7*) PD, or parkinsonism with atypical features (e.g. *PARK9*) [1]. Functional characterization of these genetic variants has provided important insights into the molecular mechanisms underlying PD and elucidated novel targets for therapeutic intervention.

Whole-exome sequencing (WES) is a powerful tool for gene discovery in pedigrees that are not sufficiently large for traditional linkage analysis [2] and this technique has been successful in identifying two causal genes for PD [3, 4]. In this study we present data from WES and *in vitro* functional analyses that demonstrate that a missense mutation (p.G192R) in the *RAB39B* gene is the causative variant in a multi-incident family with clinically typical PD.

## Results

**Fig. 1:**
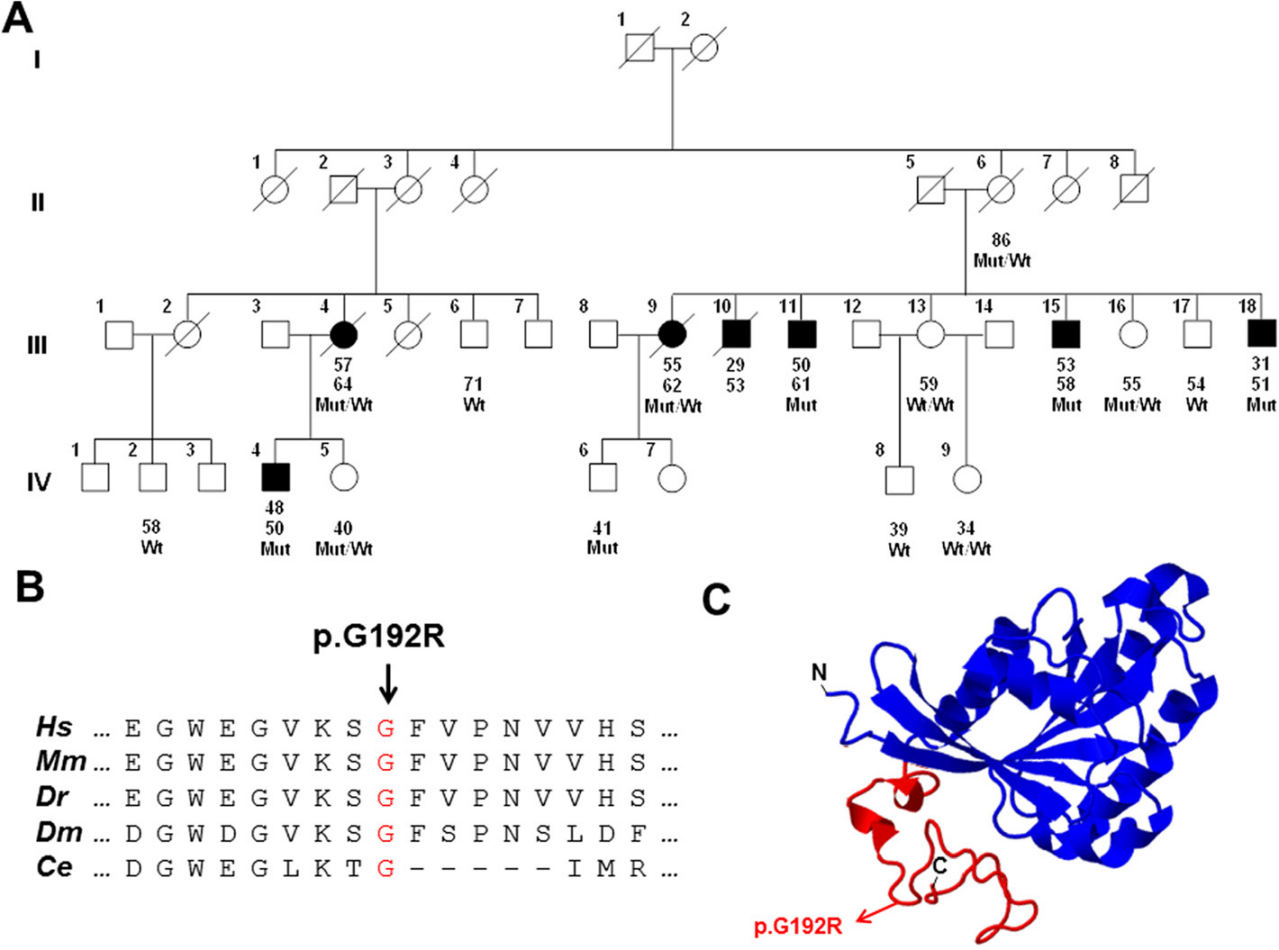
Identification of the *RAB39B* p.G192R mutation by whole exome sequencing in a multigenerational kindred with Parkinson’s disease. **a** Pedigree diagram; individuals affected with Parkinson’s disease are represented with black symbols, unaffected individuals with open symbols. Age at onset is indicated immediately below each symbol, followed by age at last clinical evaluation. Wt = wild type; Mut = mutation (p.G192R). **b** Multispecies protein sequence alignment. *Hs* = *Homo sapiens*; *Mm* = *Mus musculus*; *Dr* = *Danio rerio* (zebrafish); *Dm* = *Drosophila melanogaster*; *Ce* = *Caenorhabditis elegans*. **c** Predicted protein structure of RAB39B using Protein Homology/analogY Recognition Engine V 2.0 (Phyre^2^; http://www.sbg.bio.ic.ac.uk/phyre2/html/page.cgi?id=index) [23]; 89 % of the amino acid residues were modeled at >90 % confidence. P.G192R is located in the hypervariable C-terminal domain which is depicted in red

We studied a U.S. family of European origin in which 7 individuals (5 males and 2 females) were affected and met UK PD Society Brain Bank clinical diagnostic criteria for PD [5] (Fig. 1a). The clinical characteristics of the affected family members are provided in Table 1. None of these individuals displayed atypical findings on neurological examination but two of them (III-15 and IV-4) had mild intellectual disability since childhood. DNA was available for 6 affected and 10 unaffected members of the family. We performed WES on 4 affected (III-4, III-9, III-11, and III-18) and 1 unaffected (III-13) family members. We filtered out all variants with a frequency >1 % in 515 controls from the NHLBI Exome Sequencing Project [6, 7] or that failed to meet the quality thresholds of the Genome Analysis ToolKit (GATK) “Best Practices” [8]. We identified three nonsynonymous variants that passed all filters, segregated with PD among the 5 individuals who underwent WES, and were confirmed by Sanger sequencing: *USP1* c.573G > A (p.M191I), *MVP* c.2594G > T (p.G865V), and *RAB39B* c.574G > A (p.G192R) (Table 2). We then genotyped these three variants in all remaining family members and found that only *RAB39B* p.G192R was present in all six affected subjects. Furthermore, *RAB39B* p.G192R was not observed among 87,725 X chromosomes successfully sequenced for *RAB39B* in the Exome Aggregation Consortium database (ExAC; http://exac.broadinstitute.org). The amino acid G192 is highly conserved across species (Fig. 1b) and this mutation is predicted to be deleterious as evidenced by a Combined Annotation Dependent Depletion (CADD) [9] score of 29.4.

**Table 1:** Clinical characteristics of the affected members of the pedigree

Characteristic	Patient						
	III-4	III-9	III-10	III-11	III-15	III-18	IV-4
Sex	F	F	M	M	M	M	M
Age at onset, yr	57	55	29	50	53	31	48
Age at last evaluation, yr	64	62	53	61	58	51	50
Age at death, yr	70	64	56	─	─	─	─
Bradykinesia	Y	Y	Y	Y	Y	Y	Y
Rigidity	Y	Y	Y	Y	Y	Y	Y
Resting tremor	Y	N	Y	Y	Y	Y	Y
Postural instability	Y	N	Y	Y	N	Y	Y
Unilateral onset	Y	Y	Y	Y	Y	Y	Y
Levodopa response	Y	Y	Y	Y	Y	Y	NT
Levodopa-induced dyskinesia	N	Y	Y	Y	N	Y	─
Mild, lifelong intellectual disability	N	N	N	N	Y	N	Y
Hoehn and Yahr stage^a^	4	3	4	2.5	2	5	2.5
MDS-UPDRS III score^a^	NA	24	NA	26	19	52	11

MDS-UPDRS III = Movement Disorder Society Unified Parkinson’s Disease Rating Scale Part III; NA = not available; NT = no trial

^a^Determined at last evaluation

**Table 2:** Candidate variants identified by whole exome sequencing

Gene	Position (hg19)	Transcript	dbSNP	Variation		Allele frequency (%)^a^	CADD score	Segregation^b^
				Nucleotide	Amino acid			
*USP1*	Chr1:62910424	NM_001017415.1	─	c.573G > A	p.M191I	0	27.1	5/6
*MVP*	Chr16:29859222	NM_005115.4	rs151174471	c.2594G > T	p.G865V	0.07	7.19	5/6
*RAB39B*	ChrX:154490156	NM_171998.2	─	c.574G > A	p.G192R	0	29.4	6/6

CADD Combined Annotation Dependent Depletion

^a^Frequency among chromosomes successfully sequenced for *USP1* (n = 111,418), *MVP* (n = 121,248), and *RAB39B* (n = 87,725) in the Exome Aggregation Consortium database (http://exac.broadinstitute.org)

^b^Number of affected individuals with the variant/total number of affected individuals with genotypes in the pedigree

We then screened for *RAB39B* p.G192R in 2 cohorts of PD patients from the Parkinson’s Genetic Research Study (PaGeR). Cohort I was comprised of 203 “multiplex” families ascertained from across the U.S. (mean age at onset of probands, 57.3 years; male, 61.9 %) in which at least 2 individuals with PD were enrolled. Cohort II included 1298 unrelated PD patients (mean age at onset, 59.1 years; male, 69.2 %) enrolled primarily at movement disorder clinics in the Pacific Northwest regardless of family history. *RAB39B* p.G192R was not found in any other PD patients across PaGeR cohorts I and II (total n = 1501). We also sequenced the entire *RAB39B* coding region in the subset of patients from both PaGeR cohorts (n = 587) who reported a family history of PD and discovered two additional mutations, c.428C > G (p.A143G) and c.624_626delGAG (p.R209del). Neither of these mutations were present in the ExAC dataset and both are predicted to be deleterious *in silico* with CADD scores of 21.8 (p.A143G) and 20.4 (p.R209del). Each mutation was present in a single family but neither of the two pedigrees was sufficiently informative to assess segregation with disease (Additional file 1: Figure S1).

**Fig. 2:**
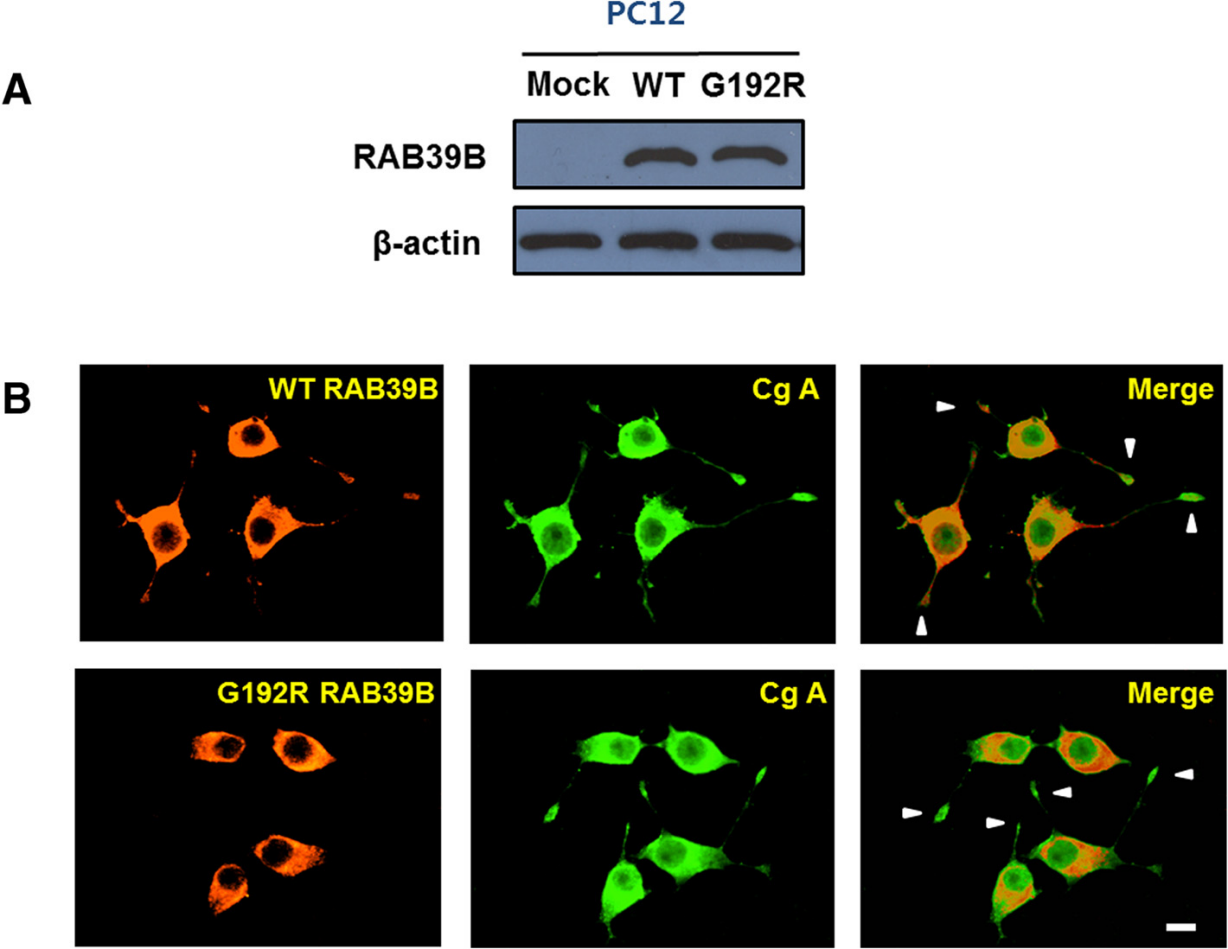
Effect of p.G192R on RAB39B expression and localization in PC12 cells. **a** Western blot of rat pheochromocytoma (PC12) cells transfected with a vector that was empty (mock), or contained a wild-type or mutant (p.G192R) construct. There was no difference in protein expression between wild-type and mutant RAB39B. **b** Immunofluorescent microscopy of nerve growth factor-differentiated PC12 cells transfected with wild-type or mutant (p.G192R) myc-tagged RAB39B. The wild-type protein is visualized throughout the cell bodies and neuritic processes, together with the vesicular marker chromogranin A, whereas the mutant protein is largely confined to cell bodies. Arrowheads indicate terminal regions of neurites. The scale bar corresponds to 10 μm

**Fig. 3:**
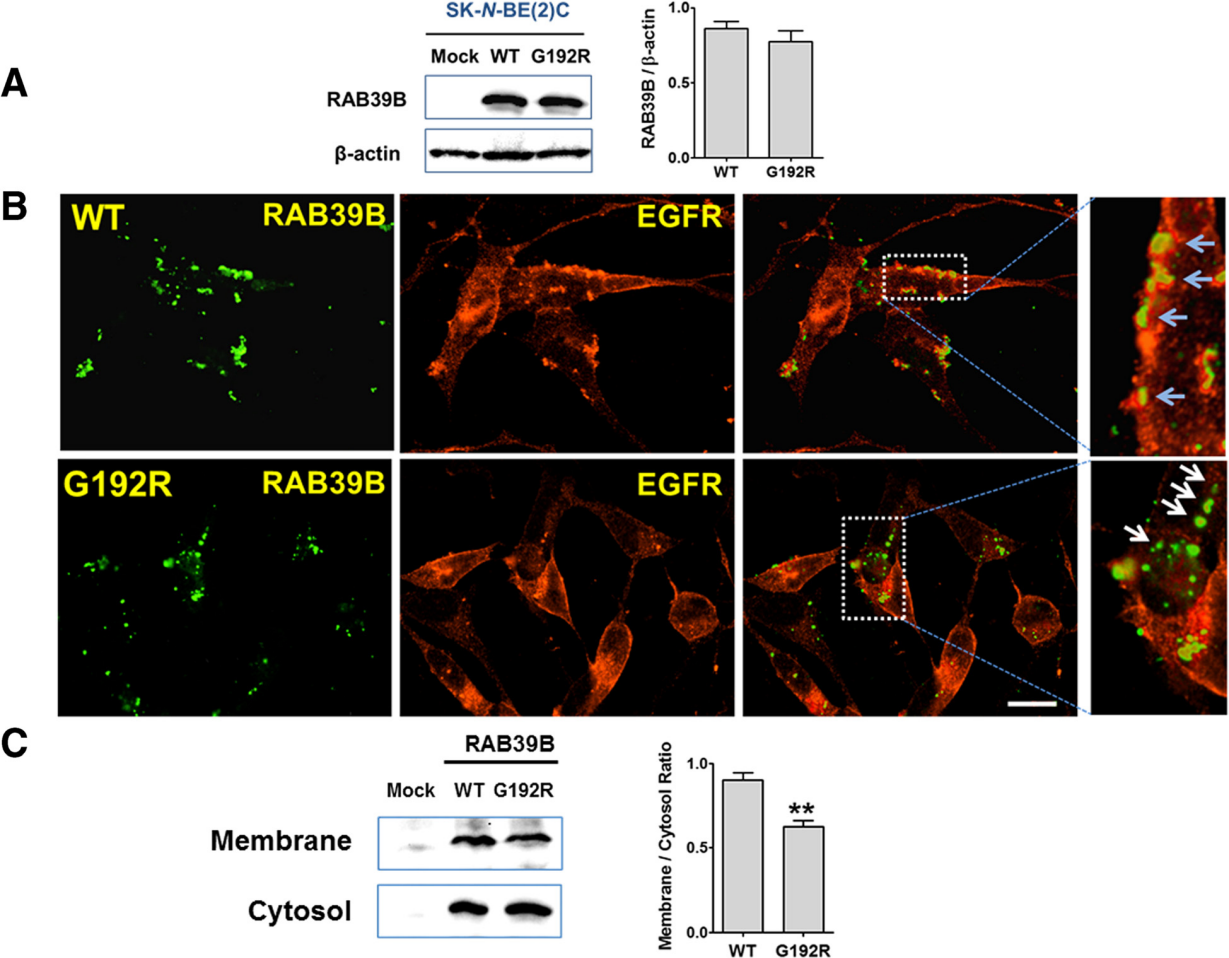
Effect of p.G192R on RAB39B expression and localization in SK-N-BE(2)C cells. **a** Western blot of human neuroblastoma (SK-N-BE(2)C) cells transfected with a vector that was empty (mock), or contained a wild-type or mutant (p.G192R) construct. There was no significant difference in protein expression between wild-type and mutant RAB39B (Student's *t*-test, *p* > 0.05. **b** Immunofluorescent microscopy of retinoic acid-differentiated SK-N-BE(2)C cells transfected with wild-type or mutant (p.G192R) GFP-tagged RAB39B. Wild-type protein was seen within the cytoplasm and at the plasma membrane where it co-localized with epidermal growth factor receptor (EGFR; blue arrows). Although mutant RAB39B protein was also apparent in the cytoplasm, it less often co-localized with EGFR (white arrows). The scale bar corresponds to 10 μm. **c** Immunoblot analysis of fractionated protein extracts performed on SK-N-BE(2)C cells transfected with a vector that was empty (mock), or contained GFP-tagged wild-type or mutant RAB39B. Double asterisks indicate a significant difference (*p* < 0.01) by Student’s *t*-test (n = 3 replicates)

We then investigated the effects of *RAB39B* p.G192R *in vitro*. In PC12 and SK-N-BE(2)C cells transfected with mutant and wild-type constructs there was no substantial difference in RAB39B protein expression (Figs. 2a and 3a). In NGF-differentiated PC12 cells wild-type RAB39B protein was visualized throughout the cytoplasm of cell bodies and neuritic processes (Fig. 2b), and co-localized with the vesicular marker chromogranin A. However, mutant (p.G192R) RAB39B was largely restricted to cell bodies with negligible amounts of protein evident in neuritic processes. In these experiments there was robust expression of both mutant and wild type RAB39B protein, but cellular phenotype can sometimes differ based on the level of transgene over-expression [10]. Thus we used an alternate vector and method of transfection to over-express RAB39B at lower levels in retinoic acid-differentiated SK-N-BE(2)C cells. Wild type RAB39B protein was frequently visualized within the cytoplasm and at the plasma membrane (co-localized with EGFR; Fig. 3b). However, while mutant RAB39B protein was also abundant in the cytoplasm, it was less frequently observed at the plasma membrane. To quantify these findings we performed immunoblot analysis of fractionated protein extracts from these cells (Fig. 3c). The proportion of membrane-bound to cytosolic RAB39B protein was significantly lower in cells expressing mutant protein than wild type protein (*p* < 0.01).

## Discussion

In the present study we provide strong evidence that the missense mutation p.G192R in *RAB39B* results in clinically typical, levodopa responsive PD. The pattern of inheritance is X-linked dominant with reduced penetrance among females. Age at onset varied by nearly three decades and was lower in males. A single male mutation carrier was unaffected at age 41. Since his current age is below the age at onset observed for 3 of the 5 affected males in the pedigree, he might well become symptomatic over time. Alternatively, it is possible that the presence of other genetic or environmental factors are necessary for p.G192R to achieve full penetrance.

*RAB39B* was first linked to human disease in 2010 when a nonsense and a splice site mutation in the gene were shown to cause mental retardation, sometimes accompanied by epilepsy and autism spectrum disorder, in the male members of two families [11]. Subsequently, duplications and triplications of a genomic region containing *RAB39B* were discovered in males from three families with a similar phenotype [12]. Recently, two families were reported with early onset parkinsonism in males and a missense mutation (p.T168K) or a complete deletion of *RAB39B* [13]. An autopsy of one such subject showed dopaminergic neuron loss in the substantia nigra and widespread Lewy body pathology. However, affected individuals all had features that were atypical for PD including intellectual disability, macrocephaly, and in the majority of cases, a lack of response to levodopa. In contrast, most individuals in our family displayed a classical PD phenotype, with the exception of two affected males who had mild intellectual disability, and both males and females were affected.

The explanation for the wide range of phenotypes associated with *RAB39B* mutations is not entirely clear. Previously reported mutations result in either overexpression of wild-type protein [12] or a complete loss of protein expression [11, 13]. However, the mutation discovered in our family (p.G192R) did not substantially change the overall amount of RAB39B protein expressed in PC12 or SK-N-BE(2)C cells. Instead, p.G192R appears to alter intracellular localization as the mutant protein did not properly migrate to the neuritic processes of NGF-differentiated PC12 cells and to the plasma membrane in SK-N-BE(2)C cells. This is consistent with the structural location of p.G192R based on current knowledge of Rab proteins. RAB39B is one of over 60 members of the human Rab GTPase family [14]. Rab GTPases act as molecular switches, cycling between active (GTP-bound) and inactive (GDP-bound) states to regulate intracellular vesicular trafficking in a temporally and spatially sensitive manner [15]. The C-terminus of Rab proteins contains a hypervariable domain (HVD) of 35-40 amino acids which through interactions with effector proteins plays a major role in targeting each Rab to the appropriate intracellular location [14]. The HVD has a high content of helix-breaking proline and glycine residues which contribute to the extended structure that allows for necessary protein interactions [16]. Because p.G192R is located within the HVD (Fig. 1c) and eliminates one such glycine moiety the mutation might disrupt proper targeting of RAB39B by inhibiting binding to effector molecules. Furthermore, the fact that two heterozygous females in our pedigree were affected raises the possibility that p.G192R might exert its pathogenic effects through a dominant negative mechanism. Interestingly, a dominant negative mutation in another Rab protein (Rab8) has been described that when introduced into *Xenopus laevis* induces retinal degeneration and shifts localization of the protein from Golgi and post-Golgi membranes to the cytoplasm [17].

Though many Rab proteins are well studied, the precise localization and function of RAB39B are just beginning to emerge. RAB39B is neuron-specific and plays a role in synapse formation and maintenance [11]. Recent evidence suggests that one function of RAB39B, through its interaction with protein interacting with C-kinase 1 (PICK1), is to regulate the subunit composition of heterotetrameric AMPA receptors [18]. In the absence of RAB39B, AMPA receptor composition shifts towards non GluA2-containing Ca2^+^-permeable forms. The resulting alteration in synaptic activity has been posited to underlie the lifelong intellectual disability and behavioral problems seen with loss-of-function mutations in *RAB39B*. However, the mechanism by which dysregulation of *RAB39B* leads to the selective neurodegenerative changes seen in PD is not yet known. Our discovery that p.G192R results in a “pure” PD phenotype provides an opportunity to address this important question by examining the effects of this mutation in model systems in future studies.

## Conclusions

Our findings implicate RAB39B, an essential regulator of vesicular-trafficking, in clinically typical PD. Loss-of-function mutations in this gene were previously shown to cause X-linked recessive mental retardation sometimes accompanied by autism spectrum disorder, and members of two such families were later shown to develop atypical parkinsonism. However, the phenotype in our family is classical, levodopa-responsive PD and both males and females are affected. We present *in vitro* data that provide a potential explanation for the substantial difference in phenotype between our family and those reported elsewhere. Unlike previously reported mutations which result in a complete loss of protein expression, the mutation that we have discovered (p.G192R) does not alter the overall amount of protein expressed but rather its intracellular localization. Our results suggest that dysregulation of RAB39B, which is thought to mediate vesicular transport, can lead to selective neurodegenerative changes in the absence of lifelong cognitive/behavioral dysfunction, and have important implications for future research.

## Methods

### Exome sequencing

The exome was captured using the SeqCap EZ Exome v3.0 kit (Roche/Nimblegen, Madison, WI) and sequenced with 100-base pair (bp) paired-end reads on a HiSeq2500 (Illumina, San Diego, CA) to achieve a mean coverage of 80-100X. Sequence reads were mapped to the human reference genome (GRCh37) using the Burrows-Wheeler Aligner. Variants were jointly called using the GATK HaplotypeCaller following the developer’s recommended best practices [8] (https://www.broadinstitute.org/gatk/guide/best-practices) and annotated with SnpEff [19] based on the RefSeq gene set (http://www.ncbi.nlm.nih.gov/refseq). We flagged variants failing to meet the quality thresholds described by the GATK “Best Practices” of QD (Quality by Depth) < 2.0, FS (FisherStrand; Fisher’s exact test for strand bias) > 60.0, MQ (Mapping Quality; overall mapping quality of reads averaged over all samples) < 40.0, HaplotypeScore (probability that reads flanking a variant can be explained by ≤ 2 haplotypes) > 13.0, MQRankSum (comparison of mapping qualities of reads for reference versus alternate allele) < -12.5, and ReadPosRankSum (measure of bias in position within reads between reference and alternate allele) < -8.0. We excluded alleles that occurred at a frequency >1 % in 515 unrelated white controls selected from the NHLBI Exome Sequencing Project [6, 7]. Finally, we used custom software to analyze variants that passed all filters to identify alleles that segregated with disease.

### Sanger sequencing and genotyping

Sanger sequencing was used to confirm and genotype candidate variants in all available members of the pedigree. We also sequenced the entire *RAB39B* coding region and intron-exon boundaries in the probands from PaGeR Cohort I (n = 203) and in the subset of patients from Cohort II (n = 384) who reported a family history of PD. Sequencing was performed using the Applied Biosystems Big-Dye Terminator v3.1 Cycle Sequencing Kit on an ABI PRISM 3130 genetic analyzer (Applied Biosystems, Foster City, CA) as described elsewhere [20]. Sequence data were base-called, aligned, and scanned for variation using Mutation Surveyor (SoftGenetics, State College, PA). *RAB39B* p.G192R was genotyped in the remainder of PaGeR Cohort II using a custom TaqMan assay.

### Protein expression assays

Two myc-tagged constructs encoding either wild type or mutant (p.G192R) RAB39B protein were created using the vector pcDNA 3.1/myc-His (Invitrogen Life Technologies, Carlsbad, CA). Rat pheochromocytoma (PC12) and human neuroblastoma (SK-N-BE(2)C) cells were grown and transfected with wild type or mutant constructs using either a retrovirus (pCL Vector System, Orbigen, San Diego, CA) for PC12 cells or Lipofectamine (Invitrogen) for SK-N-BE(2)C cells using previously described methods [21]. After 24 h the cells were lysed, and the lysates were subjected to 10 % sodium dodecyl sulfate-polyacrylamide gel electrophoresis and transferred to a polyvinylidene difluoride membrane (Bio-Rad Laboratories, Hercules, CA). The membranes were incubated with an anti-myc antibody (Roche Life Sciences, Branford, CT) and detection was achieved using the Novex ECL chemiluminescent substrate reagent kit (Invitrogen).

### Protein trafficking experiments

PC12 cells were transfected with a wild type or mutant (p.G192R) myc-tagged *RAB39B* construct as described in the previous section and differentiated with nerve growth factor (NGF) for 4 days. The cells were fixed in 4 % paraformaldehyde and then incubated overnight at 4 °C with rabbit anti-myc and anti-chromogranin A antibodies. SK-N-BE(2)C cells were transfected with GFP-tagged wild type or mutant *RAB39B* constructs (pEGFP-N1 vector; Clontech Laboratories, Mountain View, CA) using Lipofectamine. After retinoic acid-induced differentiation for 4 days the cells were fixed in 4 % paraformaldehyde and incubated overnight at 4 °C with anti-epidermal growth factor receptor (anti-EGFR) antibody (a plasma membrane marker; Cell Signaling Technology, Danvers, MA) using methods described elsewhere [22]. Hoechst 33342 was used for counterstaining and confocal analysis was performed using an Olympus IX81 microscope.

### Subcellular fractionation

SK-N-BE(2)C cells were transfected with GFP-tagged wild type or mutant *RAB39B* constructs as described in the previous section. After 24 h the cells were lysed and membrane and cytoplasmic fractions were prepared using the Subcellular Protein Fractionation kit for Cultured Cells (Thermo Scientific, Rockford, IL) according to the manufacturer’s recommendations. The fractionated lysates were subjected to 10 % sodium dodecyl sulfate-polyacrylamide gel electrophoresis and transferred to a polyvinylidene difluoride membrane. The membranes were incubated with an anti-GFP antibody (Santa Cruz Biotechnology). Images of western blots were captured using the ChemiDoc XRS system (Bio-Rad Laboratories) and scanned films were quantified using Image J software (http://rsb.info.nih.gov/ij/).

## Additional file

Additional file 1: Figure S1. Pedigrees with variants of unknown significance. Pedigrees in which the RAB39B (A) c.428C>G (p.A143G) and (B) c.624_626delGAG (p.R209del) variants were observed. Individuals affected with Parkinson’s disease are represented with black symbols, unaffected individuals with open symbols. Age at onset is indicated immediately below each symbol, followed by age at last clinical evaluation. Wt = wild type; Mut = mutation. (PDF 35 kb)
