# Supplementary figures and images for: The RAB39B p.G192R mutation causes X-linked dominant Parkinson’s disease
Source: Mol Neurodegener. 2015 Sep 24;10:50. doi: 10.1186/s13024-015-0045-4 (PMC4581468; doi:10.1186/s13024-015-0045-4)

**A****p.A143G**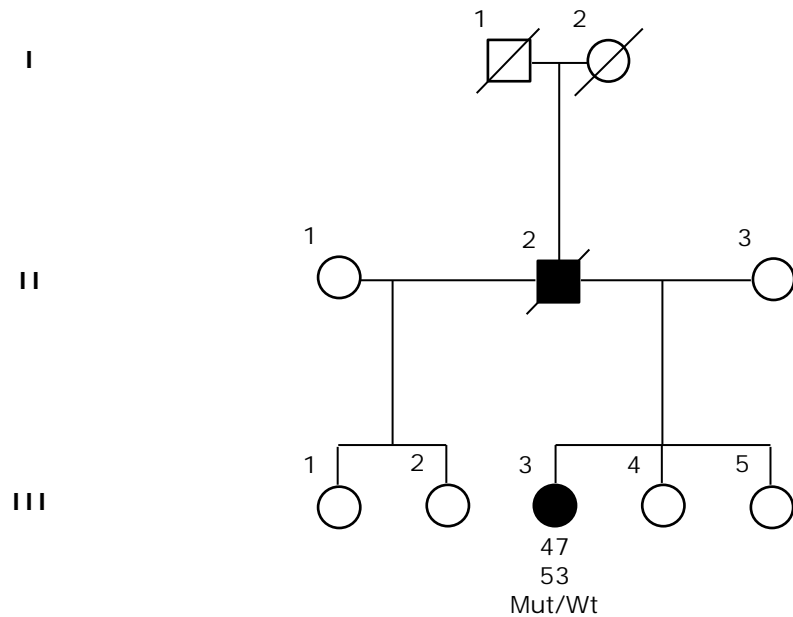**B****p.R209del**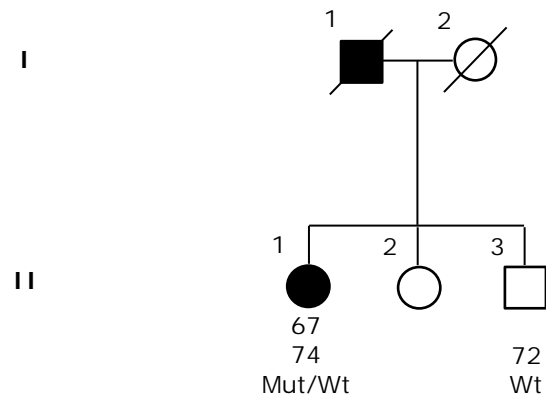

Supplement: Additional file 1: Figure S1. — Pedigrees with variants of unknown significance. Pedigrees in which the RAB39B (A) c.428C>G (p.A143G) and (B) c.624_626delGAG (p.R209del) variants were observed. Individuals affected with Parkinson’s disease are represented with black symbols, unaffected individuals with open symbols. Age at onset is indicated immediately below each symbol, followed by age at last clinical evaluation. Wt = wild type; Mut = mutation. (PDF 35 kb) [file 13024_2015_45_MOESM1_ESM.pdf]
